# The Distribution of Synonymous Codon Choice in the Translation Initiation Region of Dengue Virus

**DOI:** 10.1371/journal.pone.0077239

**Published:** 2013-10-25

**Authors:** Jian-hua Zhou, Jie Zhang, Dong-jie Sun, Qi Ma, Hao-tai Chen, Li-na Ma, Yao-zhong Ding, Yong-sheng Liu

**Affiliations:** State Key Laboratory of Veterinary Etiological Biology, Lanzhou Veterinary Research Institute, Chinese Academy of Agricultural Sciences. Lanzhou, Gansu, P.R. China; Fondazione IRCCS Policlinico San Matteo, Italy

## Abstract

Dengue is the most common arthropod-borne viral (Arboviral) illness in humans. The genetic features concerning the codon usage of dengue virus (DENV) were analyzed by the relative synonymous codon usage, the effective number of codons and the codon adaptation index. The evolutionary distance between DENV and the natural hosts (*Homo sapiens*, *Pan troglodytes*, *Aedes albopictus* and *Aedes aegypti*) was estimated by a novel formula. Finally, the synonymous codon usage preference for the translation initiation region of this virus was also analyzed. The result indicates that the general trend of the 59 synonymous codon usage of the four genotypes of DENV are similar to each other, and this pattern has no link with the geographic distribution of the virus. The effect of codon usage pattern of *Aedes albopictus* and *Aedes aegypti* on the formation of codon usage of DENV is stronger than that of the two primates. Turning to the codon usage preference of the translation initiation region of this virus, some codons pairing to low tRNA copy numbers in the two primates have a stronger tendency to exist in the translation initiation region than those in the open reading frame of DENV. Although DENV, like other RNA viruses, has a high mutation to adapt its hosts, the regulatory features about the synonymous codon usage have been ‘branded’ on the translation initiation region of this virus in order to hijack the translational mechanisms of the hosts.

## Introduction

Dengue is a common mosquito-borne *flavivirus* disease of the international public health threat. Dengue virus (DENV) can lead to a wide range of symptom from the asymptomatic state to a severe, life threatening syndrome [1], [2]. This virus is a positive-sense and single-stranded RNA virus belonging to the *flaviviridae* family, and the single reading open frame of this virus can encodes three structural proteins, including core, envelope and membrance proteins, and seven non-structural proteins namely NS1, NS2a-b, NS3, NS4a-b and NS5 [3]. DENV has four antigenically distinct serotypes, namely DENV 1, DENV 2, DENV 3 and DENV 4 [4]. Recently there have been some focuses on the genetic diversity and the evolutionary processes [5], [6], [7]. It was reported that the isolated zones of DENV play a role in influencing the evolution process of this virus [8]. By making phylogenetic tree of DENV strains derived from the open reading frame from the four genotypes, the distinct genetic divergence among the genotypes exists in the evolution processes [7]. The homology of the four genotypes of DENV are about 67–75% at the amino acid level [9]. Due to the effect of mutation from RNA virus, synonymous codons are selected with different frequencies, the feature is termed as the synonymous codon usage bias. Population genetic analyses indicate that the synonymous codon usage is influenced by an equilibrium between mutation, genetic drift and translation selection [10]. The analysis of the synonymous codon usage has been applied to investigate the relationship between mutation pressure from virus and translation selection from host [11], however, the role of the synonymous codon usage in the formation of the genetic divergence for the four genotypes has not been analyzed up to date. DENV can be transported in long distances by the hosts and *Aedes* vectors. *Aedes* mosquitoes, which serve as an important vector and spread out all over the world, can take part in the epidemic DENV emergence events, because the efficiency of the endemic cycle of DENV is greatly enhanced by the changes of the ecology and behavior of *Aedes* mosquitoes [7]. The comparison of the synonymous codon usage between virus and its natural host is an available standard to estimate the evolutionary processes and genetic features of the interesting viruses which respond or adapt to the environment of the host cells [12], [13], [14]. To date, the study has not been carried out to investigate the interaction between the virus and *Aedes* vector in the evolutionary processes at the level of the synonymous codon usage. By analysis of the similarity degree of codon usage between DENV and its hosts, this study aims to investigate the effects of its hosts on DENV at the aspect of the overall codon usage pattern.

Although evolutionary studies generally suggest that the viral genes with efficient expression represent the high codon adaptation in host cell environment, the precise fitness of viral genome associated with translationally adapted codons remains a topic of active debate [15], [16], [17], [18]. A foreign gene might be translated in the target cells, unfortunately, the interesting protein is sometimes inactive. There would be some possible reasons for the synthesis of the inactive protein, one of these is the genetic code discrepancy between the foreign gene and the host cells [19], [20], [21]. Furthermore, Tuller et al. described a general trend in the intragenic codon usage that the first 30–50 codons are, on average, translated with low efficiency [22]. Based on these interesting findings about the role of synonymous codon usage bias in gene expression mentioned above, we employed some efficacious analyzing methods to investigate the roles of the synonymous codon usage in the evolutionary processes of DENV and fitness of this virus to the two natural hosts (*Homo sapiens* and *Pan troglodytes*) and two vectors (*Aedes aegypti* and *Aedes albopictus*).

## Materials and Methods

### Coding sequences of DENV

The information of 119 strains of DENV, including 36 genotype 1, 33 genotype 2, 25 genotype 3 and 25 genotype 4, were downloaded from the GenBank of National Center for Biotechnology Information (NCBI) (http://www.ncbi.nlm.nih.gov/GenBank/) and listed in Table S1.

### Synonymous codon usage pattern and index for codon usage

To investigate the variation avoiding the confounding influence of amino acid composition of ORFs in each genotype of DENV, respectively, the relative synonymous codon usage (RSCU) values for ORF was calculated according to the published equation [23]. The three stop codons and the codons encoding for Trp and Met were excluded from the RSCU calculation. Here we employed the principal component analysis (PCA), which can reduce data dimensionality by performing a covariance analysis among 59 synonymous codons to estimate effects of viral genotypes and isolated zones on the genetic features of DENV at the level of the synonymous codon usage.

The effective number of codons (ENC) is a widely accepted measure which estimates the magnitude of the overall codon usage bias for an individual gene [24]. In addition, codon adaptation index (CAI) is also applied to quantify the magnitude of codon usage bias for an interesting gene and is a measurement of the relative adaptation of the codon usage of a gene towards the codon usage of highly expressed genes [25]. ENC and CAI value, together with the content of GC_3_ (G+C at the third synonymous position of codon) were used to estimate the role of variation of codon usage of gene in the evolutionary processes, here, we can estimate the correlation between the two major axis (*f'_1_ and f'_2_* values, which represent the synonymous codon usage of DENV, stemming from reducing data dimensionality of the PCA performance) and ENC value, CAI value, GC_3_% for the 119 ORFs of DENV, respectively. The roles of the synonymous codon usage bias and nucleotide composition in the formation of the overall codon usage of this virus in the evolutionary processes is also estimated, by means of Spearman's rank correlation performed by the SPSS 11.5.

### Estimating effects of the overall codon usage of the hosts on that of DENV

Based on codon usage frequencies of the genomes of the two primates (*Homo sapiens* and *Pan troglodytes*) and the two vectors (*Aedes aegypti* and *Aedes albopictus*) [26], the RSCU values for these organisms were also calculated for the 59 synonymous codons by the formula for RSCU value.

To estimate the effect of the overall codon usage of the hosts on that of DENV, a formula of *D(A,B)* was established to evaluate the potential role of the overall codon usage pattern of the host in the formation of the overall codon usage of DENV.
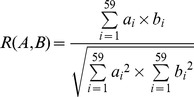


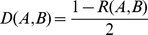
where *R(A,B)* is defined as a cosine value of an included angle between *A* and *B* special vectors representing the degree of similarity between DENV and a specific host at the aspect of the overall codon usage pattern, *a_i_* is defined as the RSCU value for a specific codon in 59 synonymous codons of DENV ORF, *b_i_* is termed as the RSCU value for the same codon of the host. *D(A,B)* represents the potential effect of the overall codon usage of the host on that of DENV, and this value ranges from zero to 1.0.

### The synonymous codon usage in the translation initiation region of DENV

To analyze the synonymous codon usage bias in the translation initiation region which are composed of the aligned codons locating in the first 20, 40, 60, 80 and 100 codon sites of the DENV ORF, respectively, we depended on a simple methods based on the previous reports [27], [28].
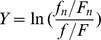
where *f_n_* is the sum of a certain synonymous codon in the specific length ranging from the start codon (AUG) to the *n^th^* codon in DENV ORF, *F_n_* is the sum of the corresponding amino acids in the given region, *f* is the sum of this synonymous codon in DENV ORF, *F* is the sum of the corresponding amino acid in the given ORF.

## Results and Discussion

Projection of the overall codon usage of DENV, by ORFs, onto the two-dimensional map by PCA can reflect the genetic diversity at the level of the synonymous codon usage. There is an interesting phenomenon that the four genotypes of DENV have an obvious genetic divergence each other, at the level of the overall codon usage (File S1), suggesting that the formation of overall codon usage in this virus might be subjected to the evolutional process of each genotype. RNA viruses are ubiquitous cellular parasites and have a strong capability to replicate and evolve rapidly [29]. The investigation of the synonymous codon usage of DENV genome revealed the evolution distinction of the four genotypes as well as their conserved evolution features and allowed for more precise and broad classification of this virus into genetically distinct groups or genotypes within DENV strains [8], [30], [31]. Based on the information of isolated zones of DENV in Table S1, no evidence of geographical limitations influencing the genetic diversity of DENV 1 and DENV 4 is found (Files S2 and S3, the information of isolated zones of DENV 2 and DENV 3 is not available to reveal the geographical limitations on the genetic diversity). This result might support the view point that with the development of urbanization, water distribution systems as well as sewer and waste management enable *Aedes* mosquitoes to reach high densities and facilitated dispersal of the DENV strains among diverse geographic regions [7] and suggest that inner factors including mutation pressure in viral genome and the interaction between virus and host might play more important roles in the formation of the overall codon usage of this virus than the extraneous factors such as geographic limitation.

It is found that the general trend of the 59 synonymous codon usage is relatively consistent among different genotypes of DENV (File S4 and Table 1). The result implies that the evolutionary processes of the four genotypes of DENV are restricted by the synonymous codon usage pattern to some degree. As for the synonymous codon usage bias for this virus, 9 under-represented codons (UCG for Ser, CCG for Pro, ACG for Thr, GCG for Ala, CGU, CGC, CGA and CGG for Arg, GGU for Gly) and 5 over-represented codons (GUG for Val, UCA for Ser, CCA for Pro, ACA for Thr, AGA for Arg) exist in DENV, in addition, GUA for Val (RSCU value< 0.6) exists in the DENV 1–3, excluding the DENV 4 (RSCU value  = 0.62) (Table 1). These data suggest that although DENV is a RNA virus with high mutation rate in its life-cycle, this virus has evolved to form a relatively stable genetic marker at some specific synonymous codon usage. In addition, there are significant correlations between the first axis (*f*'*_1_*), the second axis (*f*'*_2_*) and ENC, CAI, GC_3_%, respectively (Table 2). We found that the two index (ENC value and GC_3_%), which can reflect the role of mutation factor in the nucleotide composition of DENV ORF, have high and positive correlation with the overall codon usage pattern of this virus, while the low and negative correlation exists between the CAI value and the overall codon usage pattern. The data implies that the synonymous codon usage bias takes part in the evolutionary process of DENV. With the advent of comparative analysis for DENV ORF at the aspect of codon usage, it is possible to dissect the genetic structure of population of this virus and represent the processes governing the viral evolution.

**Table 1:** The synonymous codon usage pattern (RSCU value) of DENV and the four hosts.

C/A*	DENV 1	DENV 2	DENV 3	DENV 4	*Homo sapiens*	*Pan troglodytes*	*Aedes aegypti*	*Aedes albopictus*
UUU(F)	0.96	0.94	1.06	1.22	0.87	0.77	0.56	0.48
UUC(F)	1.04	1.06	0.94	0.78	1.13	1.23	1.44	1.52
UUA(L)	0.73	0.72	0.83	0.75	0.39	0.35	0.35	0.23
UUG(L)	1.13	1.16	1.31	1.39	0.73	0.64	1.34	1.11
CUU(L)	0.68	0.61	0.82	0.7	0.73	0.70	0.67	0.49
CUC(L)	0.70	0.95	0.93	1.00	1.21	1.35	0.81	0.87
CUA(L)	1.31	1.06	0.91	0.86	0.40	0.41	0.54	0.57
CUG(L)	1.46	1.50	1.19	1.29	2.53	2.56	2.28	2.73
AUU(I)	0.80	0.78	0.96	0.96	1.03	0.95	1.00	0.74
AUC(I)	0.81	1.04	0.74	0.87	1.52	1.57	1.59	1.86
AUA(I)	1.39	1.19	1.31	1.17	0.44	0.48	0.40	0.40
GUU(V)	0.86	0.82	0.86	0.71	0.69	0.62	1.05	0.88
GUC(V)	0.84	0.96	0.90	0.96	1.00	0.99	1.09	1.30
GUA(V)	0.58	0.59	0.59	0.62	0.42	0.36	0.60	0.51
GUG(V)	1.72	1.64	1.65	1.72	1.90	2.03	1.26	1.31
UCU(S)	1.00	0.79	0.82	1.12	1.11	1.22	0.67	0.54
UCC(S)	0.97	0.88	0.97	0.88	1.39	1.44	1.20	1.40
UCA(S)	2.17	2.07	2.15	2.08	0.84	0.80	0.68	0.48
UCG(S)	0.32	0.33	0.46	0.40	0.33	0.31	1.41	1.70
AGU(S)	0.74	0.95	0.72	0.80	0.84	0.77	0.93	0.79
AGC(S)	0.81	0.98	0.89	0.72	1.50	1.45	1.11	1.08
CCU(P)	0.64	0.74	0.87	0.71	1.12	1.09	0.67	0.35
CCC(P)	0.74	0.67	0.77	1.08	1.35	1.42	0.83	1.13
CCA(P)	2.26	2.32	2.18	1.85	1.07	0.97	1.20	1.07
CCG(P)	0.35	0.27	0.18	0.37	0.46	0.52	1.30	1.44
ACU(T)	0.73	0.64	0.63	0.67	0.94	0.85	0.80	0.64
ACC(T)	0.92	0.83	0.75	1.03	1.52	1.70	1.48	1.79
ACA(T)	1.77	2.03	2.15	1.82	1.07	1.01	0.70	0.58
ACG(T)	0.58	0.50	0.48	0.47	0.46	0.44	1.01	0.99
GCU(A)	0.91	0.97	1.13	1.16	1.09	1.09	1.09	0.99
GCC(A)	1.34	1.16	1.11	1.30	1.64	1.57	1.48	1.81
GCA(A)	1.37	1.54	1.29	1.16	0.85	0.78	0.75	0.59
GCG(A)	0.37	0.32	0.47	0.38	0.42	0.56	0.69	0.62
UAU(Y)	1.04	0.78	0.85	0.97	0.84	0.77	0.64	0.55
UAC(Y)	0.96	1.22	1.15	1.03	1.16	1.23	1.36	1.45
CAU(H)	0.91	1.05	0.89	0.99	0.81	0.80	0.84	0.75
CAC(H)	1.09	0.95	1.11	1.01	1.19	1.20	1.16	1.25
CAA(Q)	1.19	1.21	1.29	0.99	0.51	0.46	0.81	0.60
CAG(Q)	0.81	0.79	0.71	1.01	1.49	1.54	1.19	1.40
AAU(N)	0.89	0.97	0.85	0.82	0.89	0.85	0.79	0.64
AAC(N)	1.11	1.03	1.15	1.18	1.11	1.15	1.21	1.36
AAA(K)	1.33	1.27	1.16	1.25	0.82	0.81	0.79	0.58
AAG(K)	0.67	0.73	0.84	0.75	1.18	1.19	1.21	1.42
GAU(D)	0.86	0.83	0.89	0.88	0.89	0.79	1.12	0.96
GAC(D)	1.14	1.17	1.11	1.12	1.11	1.21	0.88	1.04
GAA(E)	1.23	1.39	1.17	1.25	0.81	0.68	1.15	1.11
GAG(E)	0.77	0.61	0.83	0.75	1.19	1.32	0.85	0.89
UGU(C)	1.05	1.01	1.03	0.93	0.86	0.85	0.83	0.69
UGC(C)	0.95	0.99	0.97	1.07	1.14	1.15	1.17	1.31
CGU(R)	0.30	0.39	0.25	0.18	0.51	0.43	1.36	1.50
CGC(R)	0.43	0.40	0.35	0.31	1.20	1.21	1.25	1.32
CGA(R)	0.52	0.42	0.30	0.45	0.63	0.61	1.17	0.97
CGG(R)	0.27	0.19	0.23	0.21	1.20	1.16	1.05	1.22
AGA(R)	3.12	3.41	3.32	3.13	1.20	1.29	0.64	0.58
AGG(R)	1.36	1.18	1.55	1.72	1.26	1.30	0.53	0.41
GGU(G)	0.5	0.46	0.41	0.51	0.64	0.55	1.10	1.24
GGC(G)	0.53	0.56	0.65	0.53	1.40	1.40	1.04	1.07
GGA(G)	2.34	2.25	2.09	2.05	0.98	0.90	1.49	1.21
GGG(G)	0.63	0.73	0.85	0.92	0.98	1.15	0.37	0.47

* **represents codon/amino acid.**

**Table 2:** The corrleation between axis 1, axis 2 and ENC, CAI, GC_3_%, respectively, for codon usage of DENV.

	Axis 1 (*f*'*_1_*)	Axis 2 (*f*'*_2_*)
ENC index	r = 0.697, p = 1.411×10^−18^	r = 0.562, p = 2.899×10^−11^
CAI index	r = −0.216, p = 0.018	r = −0.306, p = 0.001
GC_3_%	r = 0.826, p = 5.664×10^−31^	r = 0.248, p = 0.007

**Figure 1:**
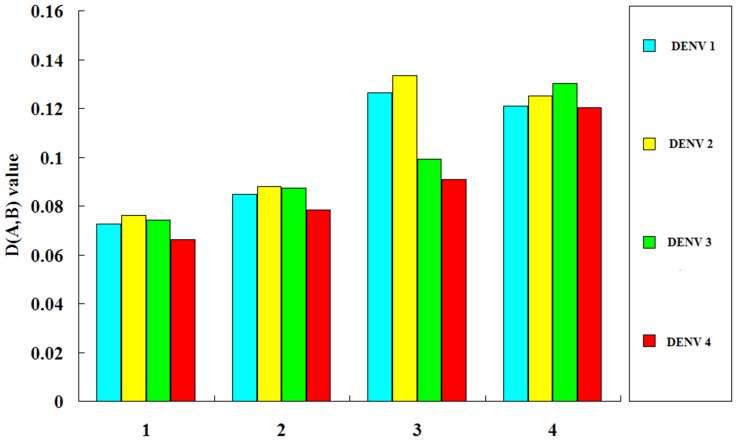
The similarity degree of the overall codon usage between DENV and the four hosts. The group 1 represents that the similarity degree of the overall codon usage between *Homo sapiens* and each genotype of DENV. The group 2 represents that the similarity degree of the overall codon usage between *Pan troglodytes* and each genotype of DENV. The group 3 represents that the similarity degree of the overall codon usage between *Aedes aegypti* and each genotype of DENV. The group 4 represents that the similarity degree of the overall codon usage between *Aedes albopictus* and each genotype of DENV.

Although the *D(A,B)* values for the four groups are not high, the index of the groups 3 & 4 (*Aedes aegypti* vs DENV and *Aedes albopictus* vs DENV) is higher than those of the groups 1 and 2 (*Homo sapiens* vs DENV and *Pan troglodytes* vs DENV), suggesting that the effect of the two *Aedes* vectors on the formation of the overall codon usage of the four genotypes is relatively stronger than that of the two primates. Some previous reports estimated the effect of synonymous codon usage of the natural hosts on that of the specific viruses, depending on the RSCU value for each synonymous codon or the adaptation of synonymous codon usage of the virus to its hosts [14], [32], [33], [34], however, these methods involving in analyzing the synonymous codon usage similarity between the virus and the hosts fail to reveal the effect of the overall codon usage of the hosts on the formation of that of the virus. Here, we do not simply analyze the similarity of the synonymous codon usage between the virus and the hosts depending on the RSCU values, but apply to estimate the similarity degree of the overall codon usage pattern comprehensively between the virus and the host by serving the 59 synonymous codons as different 59 spacial vectors. The advantage of this formula is that the comparative overall codon usage takes the place of the direct estimation of each synonymous codon usage, thus the new method avoids the situation that the variations of 59 synonymous codon usage confuse the correct estimation of the effect of the host on the virus for codon usage. As for the effects of *Aedes* vectors on the formation of the overall codon usage of DENV, the strongest effect of *Aedes aegypti* is the DENV 2, followed by the DENV 1, the DENV 3, the DENV 4, while the strongest effect of *Aedes albopictus* on the overall codon usage of DENV is the DENV 3, followed by the DENV 2, the DENV 1 and the DENV 4 (Fig. 1). As for the effects of the two primates on the formation of the overall codon usage of DENV, the strongest effect of *Homo sapiens* and *Pan troglodytes* is the DENV 2, followed by the DENV 3, the DENV 1, the DENV 4 (Fig. 1). These trends suggest that the factor of the hosts takes part in the evolutionary processes of DENV at the level of codon usage pattern. It is noted that the effects of *Pan troglodytes* on the overall codon usage pattern of DENV is stronger than that of *Homo sapiens*. The findings might imply that the *Pan troglodytes* which live in a sylvatic have a relatively stronger effect on the formation of the overall codon usage pattern of this virus than human. The potential reason is that DENV has a long history of emerging into the sylavatic transmission cycle among the primates living in the sylvatic zone, while this virus has come into the human transmission cycles which are evolutionary and ecologically distinct from those of their sylvatic ancestors [35]. In addition, the similarity of codon usage between DENV and *Aedes* vectors is generally higher than that between this virus and the primates, suggesting that the occurrence of the effect of the overall codon usage of *Aedes* vectors on that of DENV is, to some degree, prior to those of the primates. The successful human-to-human transmission depends on the secondary vector (*Aedes* mosquitoes) and the transmission cycle between mosquitoes can be performed by the vertical transmission [36], [37], [38], [39]. The models of alternating infection of arthropods and vertebrates show substantial constraints on arbovirus evolution [40], [41]. These results are similar with our analysis about the effect of the overall codon usage of *Aedes* mosquitoes on this virus is stronger than that of the primates.

For ORFs of each genotype of DENV, the preference of synonymous codons usage in the translational initiation regions with different scales was analyzed. Generally, as for each genotype of DENV, the specific synonymous codon usage preference for the amino acid has a tendency to exist in the translational initiation region. As for the translational initiation region of the DENV 1 of DENV, the synonymous codon usage preference for Ala, Gly, Pro, Thr, Arg, Leu and Ser exists in the target region. As for that of the DENV 2, the synonymous codon usage preference for Ala, Asn, Pro, Thr, Arg, Leu and Ser exist in the target region. As for that of the DENV 3, the synonymous codon usage preference for Ala, Ile, Pro, Thr, Arg, Leu and Ser exist in the target region. As for that of DENV 4, the synonymous codons for Ala, Pro, Arg and Leu exist in the target region (File S5). Recent years have seen intensive progress in reviewing protein translation regulated by codon usage bias [19], [42], [43]. Apart from the adaptation of overall codons usage of exogenous genes to the hosts, initial ORF elongation rate can play important roles in translational level of protein [16], [18], [44]. In this study, as for the synonymous codon usage of Ala, DENV 1–3 have a relatively similar synonymous codon usage tendency. GCG has a stronger tendency to exist in the 100 sites of the DENV 1–3 than that of the DENV 4; the other synonymous codons are also slightly selected in the target region, while GCC and GCG are not selected in the same region of DENV 4; GCU and GCA have a general strong tendency to exist in this region of DENV 4 (File S5). As for that of Gly, GGU in the 100 sites of DENV 1 has a strongest synonymous codon usage preference (the average R value = 1.16). Compared with others codons, this codon has slight tendency and even fails to be selected in the target region by DENV 2–4 (File S5). As for those of Val, Asn and Lys, all R values are relatively low (less than 1.0) (File S5), suggesting that codons for Val have a slight preference of synonymous codon usage in the target region. As for that of Glu, GAG has a stronger tendency to exist in the target region of DENV 2 & 4 than that of DENV 1 & 3(File S5). As for that of Ile, AUC has a stronger tendency to exist in the first 40 codon sites of DENV 3 and the first 60 codon sites of DENV 4 than the other synonymous codons (File S5). As for those of Phe and Gln, all members have a relatively slight tendency to be selected in the target region (File S5). As for that of Pro, CCG has the stronger tendency to exist in the target region of DENV 1 & 3 than the other synonymous codons, and CCU has a similar tendency to be selected in the first 40 codon sites between DENV 2 & 4 (File S5). As for that of Thr, ACG has a stronger tendency to exist in the target region of all genotypes than the other synonymous members (File S5). The first 60 codon sites of DENV 1 & 3, the first 40 codon sites of DENV 2 and the region from the 60^th^ to the 100^th^ codon sites of DENV 4 tend to select ACG highly. Turning to the synonymous codon usage for Arg, there are several synonymous codons with different preference for the 100 sites. For DENV 1, CGA, CGC and CGG have general strong existence for the translation initiation region, while GCU, AGA and AGG have general slight existence for the region; for DENV 2, CGU, CGC, CGA and CGG have different preferences in the same region, while AGA and AGG have a slight existence in the region; for DENV 3, CGU, CGC and CGG represents a stronger usage for the region, while CGA, AGA and AGG fail to do that; for DENV 4, CGC, CGA and CGG tend to be selected in the region, while CGU, AGA and AGG fail to do that (File S5). In File S5, it shows that CUG have the strongest tendency to exist in the first 20 codon sites of the translation initiation region of the four genotypes, while the others have no obvious preferences for this region. In File S5, it represents the various synonymous codon usage tendencies for the 100 sites. For DENV 1& 3, UCU has the strongest tendency to exist in the first 20 codon sites; for DENV 2, AGU has the strongest tendency for the first 20 codon sites; for DENV 4, all codons have a relatively slight existence in the given region. Some previous reports pointed out that the synonymous codon usage of the translation initiation region in the gene can play an important role in regulating the translational efficiency in other organisms [17], [45]. The optimization of the first 5–17 codons of the human *chorionic gonadotropin* gene contributes to 4- to 5-fold expression levels [46]. However, the rare codons which are highly selected in the translation initiation region of genes should be noticed, because more and more studies focus on the role of rare codons in regulating translation rate of genes [15], [47], [48], [49], [50], [51], [52], [53]. Translation initiation is an important rate-limiting step of translational efficiency, because it governs the binding and scanning of the ribosome and links the initiation and the elongation of genes. The density of ribosome which scans along coding sequence plays an important role in translation efficiency, because the traffic jam of ribosome can impair or even abort translational process [19]. In this study, those codons with high preference of usage play a role in regulating the translational efficiency of DENV ORF, and the other selections can act on the synonymous codon usage bias of the local coding sequence of this virus, except for mutation pressure from DENV.

Combining the data of File S5 with the data of Table 1, we estimated the potential roles of the synonymous codon usage preference in translation initiation region by comparing the synonymous codon usage bias between each genotype of DENV and the four hosts, respectively. We found that the under-represented codons of the four hosts and the ones of DENV are partly selected in high frequencies in the interesting region. In detail, GCG which is very lowly selected by both the four genotypes and the four hosts has a strong tendency to exist in the first 100 codon sites of DENV 1–3; GGU which is very lowly selected by DENV 1 has a strong tendency to exist in the first 40 codon sites; CCG which is very lowly selected by both the four genotypes and the two primates has a strong tendency to exist in the first 100 codon sites of DENV 1 & 3; ACG which is very lowly selected by both DENV and the two primates has a strong tendency to exist in the first 60 codon sites of DENV 1 & 3, the first 40 codon sites of DENV 2, the region from the 60^th^ to the 100^th^ codon sites of DENV 4; CGU which is very lowly selected by both DENV and the two primates has a strong tendency to exist in the region from the 20^th^ to 100^th^ codon sites of DENV 3; CGC which is very lowly selected by the virus has a strong tendency to exist in the first 100 codon sites of DENV 2 & 4, the first 20 codon sites of DENV 3 and the first 80 codon sites of DENV 1; CGA which is very lowly selected by this virus has a strong tendency to exist in the first 20 codon sites of DENV 1 & 4. CGG which is very lowly selected by this virus has a strong tendency to exist in the first 100 codon sites of DENV 1 & 3, the first 20 codon sites of DENV 2 and the region from the 60^th^ to 100^th^ codon sites of DENV 4. Among the codons which have a stronger usage bias in the translation initiation region than that of DENV ORF, some codons which correspond to low tRNA copy numbers in the two primates are strongly selected in the translation initiation region of this virus. Unfortunately, the data of tRNA copy numbers of the mosquitoes is not available and can not provide the information of the role of codons matching low tRNA copy numbers in regulating translation initiation of DENV. Based on the information about tRNA copy numbers of the two primates (http://lowelab.ucsc.edu/), these results might suggest that these codons reduce the translation initiation rate of DENV ORF by the low tRNA copy numbers of the host to some degree. The codons, which pair to the low tRNA copy numbers and are highly selected in the translation initiation region of DENV ORF, may reduce the total density of ribosome sequestering on the given coding sequence and, therefore, the optimal density of ribosome can enable them to translate the remainder at full speed. Thus if these codons in the translation initiation region reduce the probability of ribosome jamming, it would decrease the cost of gene expression at a given production level by the host. Even though DENV, like other RNA viruses, has an obvious mutation, the virus is endowed with unique regulatory features by translation selection of the host, stemming from the fact that it is ‘branded’ on a translation initiation region of this virus.

## Supporting Information

File S1 **The genetic divergence of DENV ORF at the level of codon usage.**
(TIF)

File S2 **The distribution of isolated zone of DENV 1 of DENV.**
(TIF)

File S3 **The distribution of isolated zone of DENV 4 of DENV.**
(TIF)

File S4 **The general trend of 59 synonymous codon usage for the four genotypes of DENV.**
(TIF)

File S5 **The synonymous codon usage preference for amino acids in the different lengths (the first 20 codons, the first 40 codons, the first 60 codons, the first 80 codons and the first 100 codons) of the translation initiation region of DENV ORF.**
(TIF)

Table S1 **The information about the ORF of DENV stains.**
(DOC)
