# Supplementary figures and images for: The Distribution of Synonymous Codon Choice in the Translation Initiation Region of Dengue Virus
Source: PLoS One. 2013 Oct 25;8(10):e77239. doi: 10.1371/journal.pone.0077239 (PMC3808402; doi:10.1371/journal.pone.0077239)

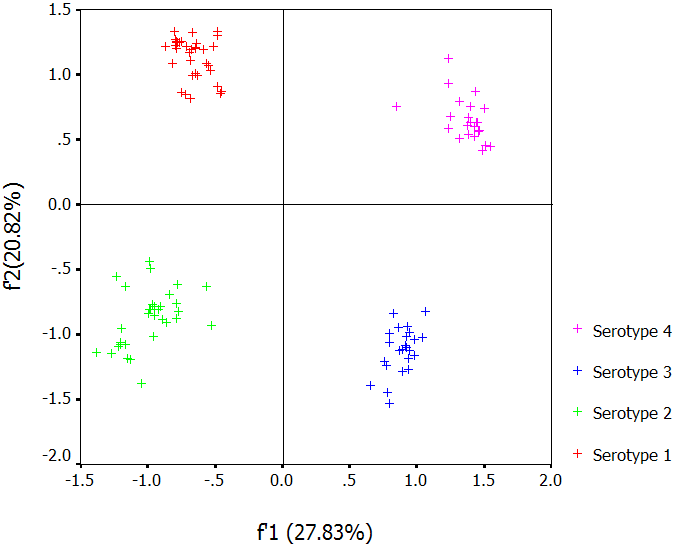

Supplement: File S1 — The genetic divergence of DENV ORF at the level of codon usage. (TIF) [file pone.0077239.s001.tif]

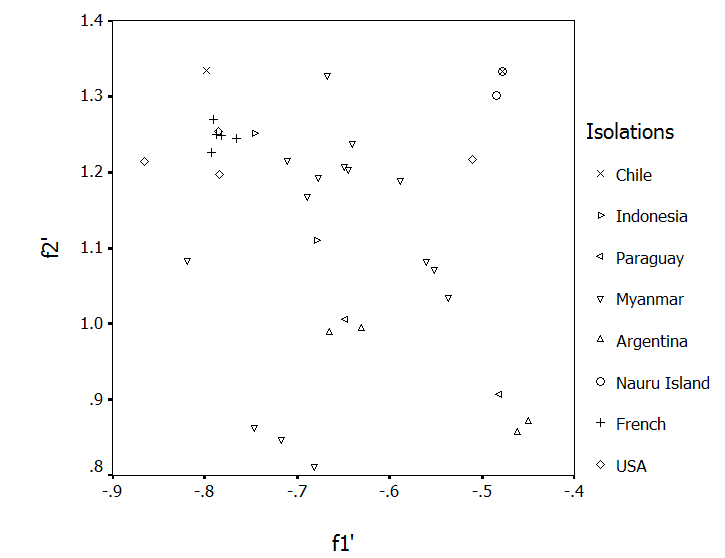

Supplement: File S2 — The distribution of isolated zone of DENV 1 of DENV. (TIF) [file pone.0077239.s002.tif]

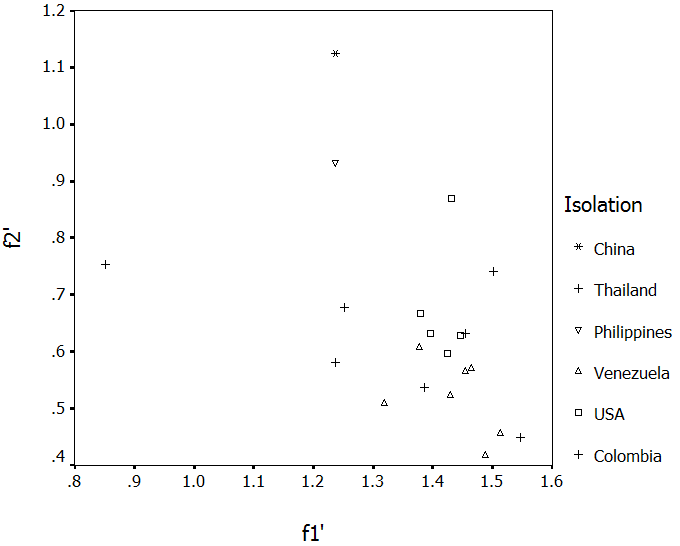

Supplement: File S3 — The distribution of isolated zone of DENV 4 of DENV. (TIF) [file pone.0077239.s003.tif]

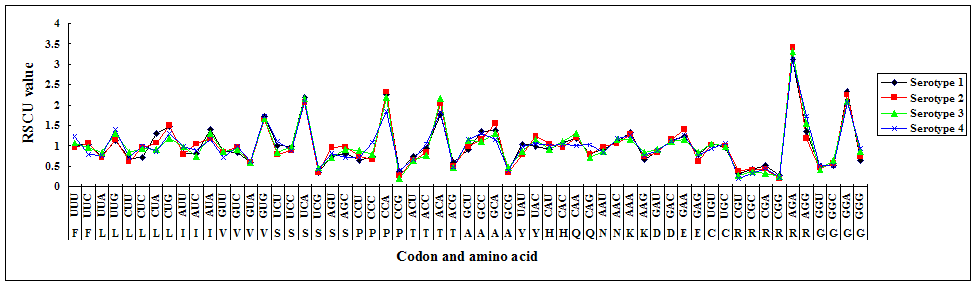

Supplement: File S4 — The general trend of 59 synonymous codon usage for the four genotypes of DENV. (TIF) [file pone.0077239.s004.tif]

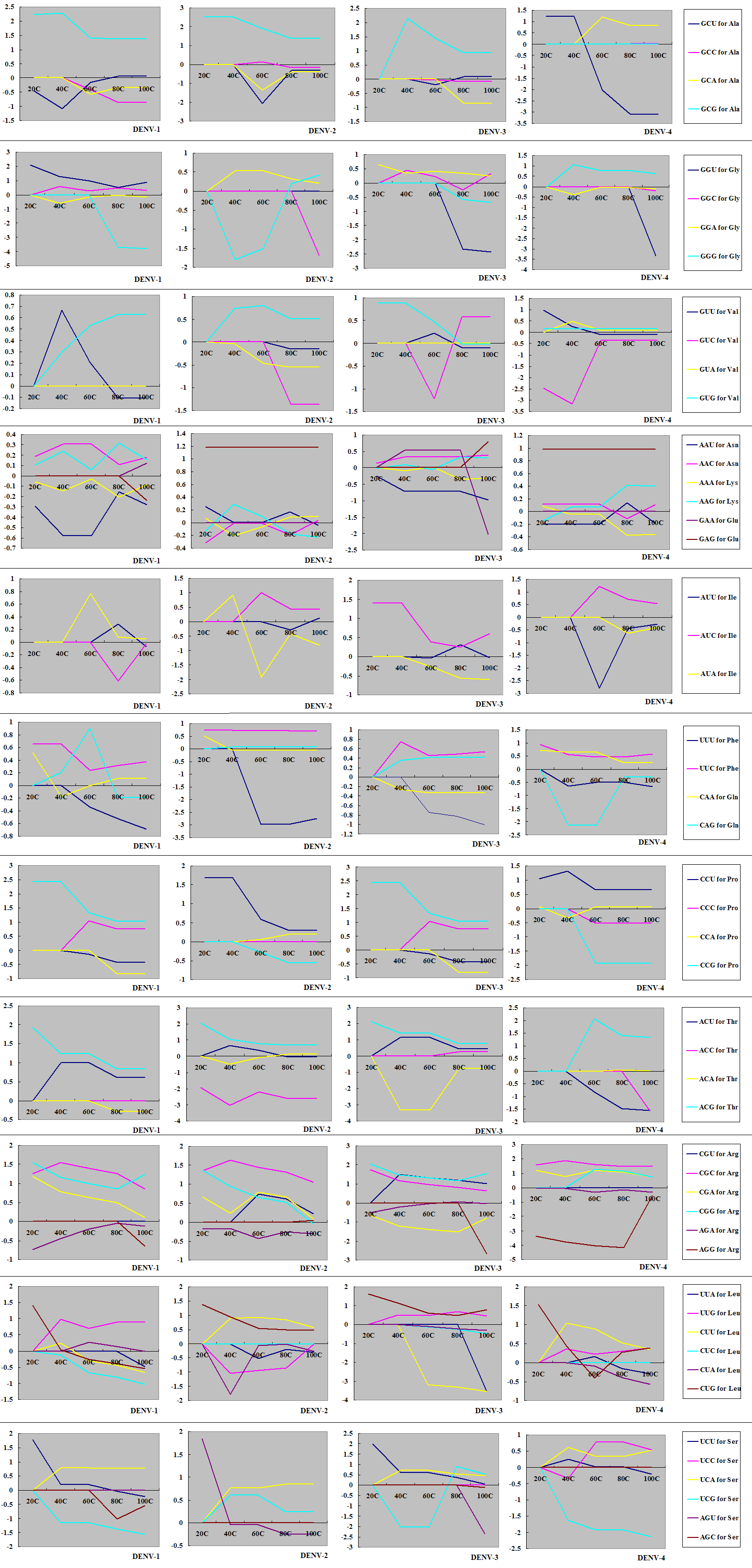

Supplement: File S5 — The synonymous codon usage preference for amino acids in the different lengths (the first 20 codons, the first 40 codons, the first 60 codons, the first 80 codons and the first 100 codons) of the translation initiation region of DENV ORF. (TIF) [file pone.0077239.s005.tif]
