# Supplementary material for: The Distribution of Synonymous Codon Choice in the Translation Initiation Region of Dengue Virus
Source: PLoS One. 2013 Oct 25;8(10):e77239. doi: 10.1371/journal.pone.0077239 (PMC3808402; doi:10.1371/journal.pone.0077239)
Supplement: Table S1 — The information about the ORF of DENV stains. (DOC) [file pone.0077239.s006.doc]

Table S1 The information about the ORF of DENV stains

| Genotype | Accession No. | Strain | Country | CAI value | ENC value | GC3% |
| --- | --- | --- | --- | --- | --- | --- |
| 1 | DQ672564 | HawO3663 | USA | 0.171 | 49.995 | 45.948 |
| 1 | DQ672563 | HawO3758 | USA | 0.171 | 50.184 | 45.977 |
| 1 | DQ672562 | HawM2540 | USA | 0.172 | 50.923 | 46.204 |
| 1 | DQ672561 | HawM3430 | USA | 0.171 | 50.188 | 45.918 |
| 1 | DQ672560 | HawM2516 | French | 0.171 | 50.177 | 45.948 |
| 1 | DQ672559 | FP1104 | French | 0.171 | 50.189 | 45.918 |
| 1 | DQ672558 | FP0908 | French | 0.171 | 50.163 | 45.889 |
| 1 | DQ672557 | FP0705 | French | 0.171 | 50.2 | 45.948 |
| 1 | DQ672556 | FP0203 | French | 0.172 | 50.199 | 45.918 |
| 1 | U88537 | NONE | Nauru Island | 0.172 | 50.496 | 46.242 |
| 1 | U88537 | NONE | Nauru Island | 0.173 | 50.307 | 46.183 |
| 1 | AF514889 | 297arg00 | Argentina | 0.169 | 49.705 | 46.006 |
| 1 | AY726555 | D1.Myanmar.31459/98 | Myanmar | 0.169 | 49.351 | 45.299 |
| 1 | AY726554 | D1.Myanmar.31987/98 | Myanmar | 0.169 | 49.018 | 45.623 |
| 1 | AY726553 | D1.Myanmar.49440/02 | Myanmar | 0.169 | 49.171 | 45.682 |
| 1 | AY726552 | D1.Myanmar.44988/02 | Myanmar | 0.169 | 49.579 | 45.859 |
| 1 | AY726551 | D1.Myanmar.44168/01 | Myanmar | 0.169 | 48.973 | 45.564 |
| 1 | AY726550 | D1.Myanmar.38862/01 | Myanmar | 0.169 | 49.373 | 45.447 |
| 1 | AY726549 | D1.Myanmar.37726/01 | Myanmar | 0.169 | 49.054 | 45.594 |
| 1 | AY722803 | D1.Myanmar.32514/98 | Myanmar | 0.167 | 49.103 | 46.566 |
| 1 | AY722802 | D1.Myanmar.23819/96 | Myanmar | 0.162 | 49.282 | 46.478 |
| 1 | AY722801 | D1.Myanmar.40568/76 | Myanmar | 0.166 | 49.064 | 46.006 |
| 1 | AY713476 | D1.Myanmar.305/01 | Myanmar | 0.168 | 48.97 | 45.741 |
| 1 | AY713475 | D1.Myanmar.206/01 | Myanmar | 0.169 | 49.335 | 45.594 |
| 1 | AY713474 | D1.Myanmar.194/01 | Myanmar | 0.169 | 49.465 | 45.830 |
| 1 | AY713473 | D1.Myanmar.40553/71 | Myanmar | 0.165 | 49.33 | 46.331 |
| 1 | AY708047 | D1.Myanmar.059/01 | Myanmar | 0.169 | 49.086 | 45.963 |
| 1 | AY206457 | 293arg00 | Argentina | 0.169 | 49.734 | 46.036 |
| 1 | AF514883 | 259par00 | Paraguay | 0.17 | 49.476 | 46.272 |
| 1 | AF514885 | 295arg00 | Argentina | 0.169 | 49.456 | 46.331 |
| 1 | AF514876 | 301arg00 | Argentina | 0.17 | 49.483 | 46.272 |
| 1 | AF514878 | 280par00 | Paraguay | 0.168 | 49.632 | 46.095 |
| 1 | AB189121 | 98901530 DF DV-1 | Indonesia | 0.175 | 49.719 | 45.152 |
| 1 | AB189120 | 98901518 DHF DV-1 | Indonesia | 0.169 | 50.332 | 46.124 |
| 1 | NC_001477 | --------- | --------- | 0.173 | 50.307 | 46.183 |
| 1 | EU863650 | CHI3336-02 | Chile | 0.171 | 50.246 | 45.800 |
| 2 | JF357907 | --------- | Nicaragua | 0.176 | 48.883 | 44.163 |
| 2 | HQ541793 | DENV-2/NI/BID-V4636/2005 | Nicaragua | 0.177 | 48.71 | 44.104 |
| 2 | EU482752 | DENV-2/NI/BID-V520/2005 | Nicaragua | 0.177 | 48.71 | 44.104 |
| 2 | FJ744725 | DENV-2/TH/BID-V2311/2001 | Thailand | 0.173 | 48.695 | 45.725 |
| 2 | GQ398314 | DENV-2/PR/13DN/1994 | Puerto Rico | 0.175 | 48.55 | 45.666 |
| 2 | GQ398272 | DENV-2/PR/49DN/1994 | Puerto Rico | 0.175 | 48.489 | 45.224 |
| 2 | GQ398268 | DENV-2/ID/1022DN/1975 | Indonesia | 0.177 | 48.617 | 46.492 |
| 2 | GQ398265 | DENV-2/SG/07K3608DK1/2008 | Singapore | 0.174 | 49.135 | 45.519 |
| 2 | HM582117 | D2/TO/UH04/1974 | Tonga | 0.178 | 48.446 | 46.122 |
| 2 | HM582110 | D2/PF/UH00/1973 | French | 0.178 | 48.447 | 46.167 |
| 2 | HM582107 | D2/AS/UH73/1972 | American | 0.179 | 48.473 | 46.093 |
| 2 | HM582102 | D2/NC/UH37/1971 | New Caledonia | 0.178 | 48.454 | 46.197 |
| 2 | HM582099 | D2/FJ/UH21/1971 | Fiji | 0.178 | 48.464 | 46.197 |
| 2 | HM488257 | DENV-2/GU/BID-V2950/2001 | Guam | 0.172 | 49.257 | 45.637 |
| 2 | AY744147 | Tonga/74 | Tonga | 0.178 | 48.37 | 46.315 |
| 2 | HM181971 | DENV-2/BR/BID-V3637/2008 | Brizal | 0.176 | 49.086 | 44.605 |
| 2 | GU131974 | DENV-2/MX/BID-V3715/2007 | Mexico | 0.178 | 48.589 | 44.251 |
| 2 | AF038403 | New Guinea C | New Guinea | 0.177 | 48.587 | 46.492 |
| 2 | AF204178 | 43 | China | 0.179 | 48.499 | 46.551 |
| 2 | AF208496 | DEN2/H/IMTSSA-MART/98-703 | Martinique | 0.174 | 48.999 | 44.399 |
| 2 | AF169680 | ThNH45/93 | ------- | 0.172 | 48.906 | 45.932 |
| 2 | U87411 | 16681 | -------- | 0.177 | 48.849 | 45.873 |
| 2 | EU081177 | D2/SG/05K3295DK1/2005 | Singapore | 0.172 | 49.01 | 45.489 |
| 2 | EF457904 | Dar Ar D75505 | Senegal | 0.164 | 48.969 | 46.728 |
| 2 | AY858036 | TB16i | Indonesia | 0.17 | 49.225 | 45.430 |
| 2 | EF105380 | Dar Ar 578 | Cote d’Ivoire | 0.166 | 49.138 | 46.669 |
| 2 | DQ645542 | 950-DF-11/12/2001 | China | 0.17 | 49.858 | 45.755 |
| 2 | DQ181798 | ThD2_0055_99 | Thailand | 0.173 | 48.968 | 45.696 |
| 2 | AY702038 | Cuba165/97 | Cuba | 0.175 | 48.741 | 44.605 |
| 2 | GU131930 | DENV-2/IPC/BID-V4270/2008 | Cambodia | 0.173 | 48.805 | 45.548 |
| 2 | GQ868555 | DENV-2/CO/BID-V3371/2005 | Colombia | 0.177 | 48.843 | 45.106 |
| 2 | GQ868595 | DENV-2/VE/BIE-V3362/1991 | Venezuela | 0.172 | 48.391 | 44.811 |
| 2 | FJ898435 | DENV-2/NI/BID-V648/2005 | Nicaragua | 0.176 | 48.741 | 44.104 |
| 3 | JF295012 | DENV-3/KH/BID-V4307/2007 | Cambodia | 0.171 | 49.522 | 46.800 |
| 3 | HQ166030 | DENV-3/NI/BID-V4738/2009 | Nicaragua | 0.171 | 49.616 | 46.476 |
| 3 | FJ644564 | ND143 | India | 0.169 | 49.618 | 47.095 |
| 3 | FJ744700 | DENV-3/VE/BID-V2186/2001 | Venezuela | 0.17 | 49.595 | 46.181 |
| 3 | AY648961 | Sleman/78 | Indonesia | 0.171 | 49.322 | 46.830 |
| 3 | AF317645 | Feb-80 | China | 0.17 | 49.652 | 46.741 |
| 3 | EU081225 | D3/SG/05K4648DK1/2005 | Singapore | 0.171 | 49.446 | 46.889 |
| 3 | EU081185 | Singapore | Singapore | 0.17 | 49.439 | 46.771 |
| 3 | AY858047 | TB16 | Indonesia | 0.17 | 49.336 | 47.184 |
| 3 | AY676350 | ThD3_0104_93 | Thailand | 0.171 | 49.512 | 46.682 |
| 3 | GU131867 | DENV-3/BR/BID-V3588/2007 | Brazil | 0.167 | 49.646 | 46.122 |
| 3 | GQ868575 | DENV-3/CO/BID-V3400/2004 | Colombia | 0.17 | 49.609 | 46.299 |
| 3 | GQ252674 | DENV-3/LK/BID-V2409/1997 | Sri Lanka | 0.172 | 49.279 | 46.417 |
| 3 | AY496877 | BDH02-7 | Bangladesh | 0.169 | 49.389 | 46.682 |
| 3 | AY744685 | PF94/136116 | French | 0.171 | 49.699 | 47.007 |
| 3 | AY496879 | PhMH-J1-97 | Philippines | 0.171 | 49.399 | 47.449 |
| 3 | FJ432743 | DENV-3/VN/BID-V1817/2007 | Viet Nam | 0.17 | 49.322 | 46.476 |
| 3 | FJ390377 | DENV-3/US/BID-V1737/1999 | USA | 0.17 | 49.454 | 46.034 |
| 3 | EU687196 | DENV-3/US/BID-V1476/2002 | USA | 0.169 | 49.582 | 46.122 |
| 3 | EU482614 | DENV-3/VE/BID-V913/2001 | Venezuela | 0.17 | 49.552 | 46.093 |
| 3 | FJ898464 | DENV-3/GY/BID-V2980/2002 | Guyana | 0.17 | 49.596 | 46.152 |
| 3 | FJ898463 | DENV-3/LC/BID-V2979/2001 | Saint Lucia | 0.17 | 49.523 | 46.122 |
| 3 | FJ898456 | DENV-3/WS/BID-V2973/1995 | Samoa | 0.171 | 49.628 | 47.036 |
| 3 | FJ898442 | DENV-3/MX/BID-V2989/2007 | Mexico | 0.168 | 49.724 | 46.682 |
| 3 | FJ882575 | DENV-3/MZ/BID-V2418/1985 | Mozambique | 0.17 | 49.482 | 46.270 |
| 4 | GU289913 | 341750 | Colombia | 0.171 | 50.712 | 47.963 |
| 4 | AF326573 | 814669 | -------- | 0.171 | 50.742 | 47.934 |
| 4 | FJ226067 | DENV-4/US/BID-V860/1994 | USA | 0.172 | 50.815 | 48.052 |
| 4 | GQ199884 | DENV-4/US/BID-V2438/1996 | USA | 0.17 | 50.667 | 47.845 |
| 4 | FJ882600 | DENV-4/US/BID-V2447/1999 | USA | 0.171 | 50.503 | 47.845 |
| 4 | FJ639737 | DENV-4/VE/BID-V2164/1998 | Venezuela | 0.172 | 50.736 | 48.170 |
| 4 | FJ024476 | DENV-4/CO/BID-V1600/1997 | Colombia | 0.173 | 50.596 | 47.816 |
| 4 | FJ639745 | DENV-4/VE/BID-V2173/1999 | Venezuela | 0.172 | 50.712 | 47.845 |
| 4 | FJ850095 | DENV-4/VE/BID-V2176/2000 | Venezuela | 0.172 | 50.864 | 48.052 |
| 4 | FJ639773 | DENV-4/VE/BID-V2206/2001 | Venezuela | 0.172 | 50.818 | 47.963 |
| 4 | GQ868581 | DENV-4/CO/BID-V3408/2001 | Colombia | 0.174 | 50.842 | 48.229 |
| 4 | FJ182016 | DENV-4/VE/BID-V1158/2007 | Venezuela | 0.172 | 50.914 | 47.786 |
| 4 | AY762085 | Singapore 8976/95 | UNKONWN | 0.171 | 51.092 | 48.007 |
| 4 | GQ868594 | DENV-4/PH/BID-V3361/1956 | Philippines | 0.175 | 50.9 | 48.524 |
| 4 | AY618993 | ThD4_0734_00 | Thailand | 0.175 | 51.087 | 48.259 |
| 4 | AF289029 | B5 | China | 0.179 | 50.634 | 48.377 |
| 4 | FJ882592 | DENV-4/VE/BID-V2501/2008 | Venezuela | 0.172 | 50.909 | 47.786 |
| 4 | GQ868585 | DENV-4/CO/BID-V3412/2005 | Colombia | 0.172 | 50.856 | 48.022 |
| 4 | GQ868584 | DENV-4/CO/BID-V3411/2004 | Colombia | 0.171 | 50.914 | 47.934 |
| 4 | EU854295 | DENV-4/US/BID-V1083/1986 | USA | 0.171 | 50.503 | 47.845 |
| 4 | GQ199880 | DENV-4/US/BID-V2431/1995 | USA | 0.173 | 50.96 | 47.757 |
| 4 | EU854297 | DENV-4/US/BID-V1094/1998 | USA | 0.17 | 50.677 | 48.022 |
| 4 | FJ639748 | DENV-4/VE/BID-V2177/2000 | Venezuela | 0.172 | 50.718 | 47.757 |
| 4 | AY618990 | ThD4_0348_91 | Thailand | 0.171 | 51.339 | 48.701 |
| 4 | FJ639745 | DENV-4/VE/BID-V2173/1999 | Venezuela | 0.172 | 50.712 | 47.845 |
